# Spline-Based Dense Medial Descriptors for Lossy Image Compression

**DOI:** 10.3390/jimaging7080153

**Published:** 2021-08-19

**Authors:** Jieying Wang, Jiří Kosinka, Alexandru Telea

**Affiliations:** 1 Faculty of Science and Engineering, University of Groningen, 9747 AG Groningen, The Netherlands; j.kosinka@rug.nl; 2 Department of Information and Computing Science, Utrecht University, 3584 CC Utrecht, The Netherlands; a.c.telea@uu.nl

**Keywords:** medial descriptors, image compression, B-splines, super-resolution

## Abstract

Medial descriptors are of significant interest for image simplification, representation, manipulation, and compression. On the other hand, B-splines are well-known tools for specifying smooth curves in computer graphics and geometric design. In this paper, we integrate the two by modeling medial descriptors with stable and accurate B-splines for image compression. Representing medial descriptors with B-splines can not only greatly improve compression but is also an effective vector representation of raster images. A comprehensive evaluation shows that our Spline-based Dense Medial Descriptors (SDMD) method achieves much higher compression ratios at similar or even better quality to the well-known JPEG technique. We illustrate our approach with applications in generating super-resolution images and salient feature preserving image compression.

## 1. Introduction

With the development of the Internet and multimedia, people create and transmit images of increasing resolution and size. As such, the demand for efficient *image compression* is growing. Within the many methods for this task, a particular class focuses on encoding images represented as threshold sets in luminance space [1] by using their medial axis transforms (MATs), which are already well known for binary image analysis, matching, and retrieval [2]. Recently, Wang et al. [3] exploited this encoding scheme and presented Compressing Dense Medial Descriptors (CDMD) for lossy image compression. Qualitative and quantitative evaluation has shown that CDMD achieves higher compression at similar quality compared to the well-known JPEG technique for specific image types. However, CDMD strongly depends on the image type, and the gains vs. JPEG are limited.

Separately from the above, the recent Spline Medial Axis Transform (SMAT) method has proposed a compact and accurate representation of MATs [4]. In this paper, we join the strengths of the CDMD representation of images using MATs with the SMAT representation of MATs to propose Spline-based Dense Medial Descriptors (SDMD), a method for efficient and accurate encoding of grayscale and color images. The contributions of our work are as follows:

- *Novelty*: Our method is, to our knowledge, the first approach to encode *color* images with B-spline-based MATs;
- *Generality*: SDMD can directly handle any raster image of any resolution;
- *Scalability*: End-to-end, our method can encode (and decode) megapixel images in a few seconds on a commodity PC featuring a modern graphics processing unit (GPU);
- *Evaluation*: We show that SDMD has good performance (compression ratio and quality) on a wide set of natural and synthetic color images of different sizes;
- *Applications*: We show that SDMD enables additional applications besides compression, such as generating super-resolution images and compression that preserves salient features.

The rest of the paper is organized as follows. Section 2 outlines related work regarding the CDMD method, the SMAT representation, and image compression. Section 3 details our SDMD method. Section 4 presents and evaluates the obtained results. Section 5 presents applications of SDMD. Section 6 discusses our proposal. Finally, Section 7 concludes the paper.

## 2. Related Work

**Figure 1:**
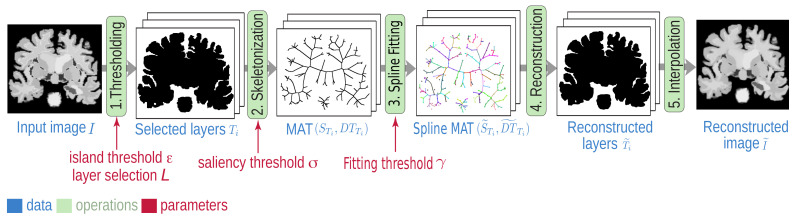
Spline-based Dense Medial Descriptors (SDMD) pipeline with free (user controlled) parameters in red.

The proposed SDMD method (Figure 1) combines the strengths of two separate methods: the CDMD method for image representation by MATs of threshold sets (Figure 1, steps 1, 2, 5) and the SMAT method for representing MATs by B-splines (Figure 1, steps 3 and 4). We next detail the CDMD and SMAT methods in Section 2.1 and Section 2.2, respectively. We position our SDMD method vs. other compression methods in Section 2.3.

### 2.1. CDMD Method

Given an input image $I:R^{2}\to \left[0,255\right]$, the CDMD method [3] first divides it into *n* (256 for 8-bit images) threshold sets or layers $T_{i}=\left\{x\in R^{2}\;|\;I\left(x\right)\geq i\right\},0\leq i<n$ (Figure 1, step 1). During thresholding, small-size islands can appear in the layers $T_{i}$ due to local intensity variations (noise). CDMD removes the islands whose size is smaller than a fraction ε of $|T_{i}|$, i.e., those which contribute little to the image *I*. Next, CDMD selects a desired number of layers L<255 to represent *I*, based on the observation that many layers contribute little to the description of *I*. For details on how this is done, we refer to the CDMD paper [3].

For the selected *L* layers $T_{i}$, CDMD next computes their medial axis transforms $\left(S_{T_{i}},DT_{T_{i}}\right)$, where

(1) $$DT_{T_{i}}\left(x\right)=\min_{y\in \partial T_{i}}∥x-y∥$$

is the distance transform $DT:T_{i}\to R^{+}$ [5] of the boundary $\partial T_{i}$ of the (binary) image of layer $T_{i}$, and

(2) $$S_{T_{i}}=\{x\in T_{i}|\exists f_{1}\in \partial T_{i},f_{2}\in \partial T_{i},∥x-f_{1}∥=∥x-f_{1}∥=DT_{T_{i}}\left(x\right)\}$$

is the medial axis, or skeleton, of $T_{i}$.

MAT computation is a well-studied technique [6,7,8,9,10,11]. CDMD uses the GPU implementation in [12] for this, which is pixel-exact and linear in the number of pixels in $T_{i}$ [13,14]. However, the generated skeletons $S_{T_{i}}$ can contain many so-called spurious branches, which take significant space but contribute little to encoding $T_{i}$. Hence, CDMD regularizes the skeletons $S_{T_{i}}$ by removing all their pixels $x\in S_{T_{i}}$ which have a so-called *saliency* value [15] below a user-specified threshold σ>0. Saliency is defined as

(3) $$\sigma \left(x\right)=\frac{\rho (x)}{DT_{T_{i}}\left(x\right)},$$

where ρ(x) denotes the fraction of the boundary $\partial T_{i}$ that the skeletal pixel x encodes [16]. Saliency-based regularization removes spurious skeleton branches corresponding to small-scale boundary perturbation but keeps intact branches that correspond to important boundary features such as large-scale corners, as shown in related work [3,15].

From the regularized (simplified) MAT $\left(S_{T_{i}},DT_{T_{i}}\right)$, one can reconstruct a simplified version $\tilde{T_{i}}$ of each layer $T_{i}$ as the union $\cup_{x\in S_{T_{i}}}B\left(x,DT_{T_{i}}\left(x\right)\right)$ of discs *B* centered at pixels x of the simplified skeletons $S_{T_{i}}$ and with radii given by the distance transform $DT_{T_{i}}\left(x\right)$. An approximation of the original image *I* can then be obtained by drawing all reconstructed layers $\tilde{T_{i}}$ atop each other in increasing order of luminance *i*. To further reduce banding artifacts between two consecutive layers $\tilde{T_{i}}$ and ${\tilde{T}}_{i+1}$, CDMD performs an interpolation operation based on blending with weights determined by distance transforms $DT_{\tilde{T_{i}}}$ and $DT_{{\tilde{T}}_{i+1}}$.

CDMD’s main value was in showing that a grayscale or color image can be faithfully encoded by a set of per-layer MATs. However, CDMD’s storage costs are prohibitive: if we want a very high-quality reconstruction, storing the pixel-representations MATs for *L* layers is barely more efficient than storing the original image encoded by these.

### 2.2. SMAT Method

The MATs extracted by CDMD (Section 2.1) provide an accurate way to encode an image *I*. However, they are quite expensive, as one has to store *L* MATs, each represented as a set of pixels with 2D locations and DT values. The issue of compactly encoding MATs has received attention in areas outside image representation, most notably for encoding MATs for *binary* shapes. In particular, representing MATs with splines was found to be good for data compression as storing spline control points is less costly than storing all MAT points. Yushkevich et al. [17] first proposed to fit the MAT with cubic B-splines for statistical shape analysis. Zhu et al. improved this by *automatically* computing a compact spline representation of the MAT of a 2D binary shape [18]. However, this approach handles only *vector* shape representations, i.e., only works with the Voronoi-based MAT method of [19]. In contrast, the SMAT method of Wang et al. [4] used *raster* representations for $T_{i}$, $S_{T_{i}}$, and $DT_{T_{i}}$, fitting $S_{T_{i}}$ and $DT_{T_{i}}$ with B-splines. SMAT is directly applicable to any binary image $T_{i}$ and also can use the computationally efficient methods for extracting the MAT [12]. As such, we further adopt SMAT in our pipeline.

SMAT applies a least-squares algorithm [20] to fit every MAT branch in the 3D space $\left(S_{T_{i}}\times DT_{T_{i}}\right)$ with a B-spline. For a user-provided approximation error γ between the MAT and the B-splines, SMAT finds the fitting scheme with the minimal number of B-spline control points required. Each control point $c_{j}=\left(p_{j},DT_{T_{i}}\left(p_{j}\right)\right)\in R^{3}$ consists of a 2D position $p_{j}$ and its corresponding DT value. Hence, instead of storing all MAT pixels (as CDMD does), SMAT stores only a smaller set of control points. From these, SMAT rasterizes the B-splines using de Casteljau’s algorithm [21]. Thus, the rasterized B-splines give a pixel-based representation of the MAT. From this representation, a layer $\tilde{T_{i}}$ is reconstructed by the disc-union method described earlier in Section 2.1. For full implementation details, we refer to [4].

Summarizing: The CDMD method faithfully (but not compactly) represents a grayscale or color image using pixel-based MATs for several layers. The SMAT method compactly and faithfully encodes a MAT for a single layer using B-splines. Our SDMD proposal next combines the two to faithfully *and* compactly represent a grayscale or color image.

### 2.3. Image Compression Methods

Obviously, representing and compressing images can be done with many other methods than medial ones such as CDMD and SMAT. Many image compression methods have been proposed in the literature, which can be divided into two main classes: lossless and lossy ones. Lossy compression has seen great interest due to its particularly high compression ratio (CR) while maintaining visual quality. In the past few decades, countless lossy compression approaches have been proposed. In the early days, transform domain coding dominated, which includes the well-known discrete cosine transform, discrete wavelet transform, and discrete Fourier transforms [22,23,24,25]. However, all these methods divide the image into non-overlapping blocks for processing. When a high compression rate is desired, the results tend to show specific artifacts such as blocking or banding.

In recent years, Deep Neural Network (DNN) methods have attracted increasing interest due to their high compression rate and good quality. Important methods in this area use Recurrent Neural Networks (RNNs) [26,27,28] and also autoencoders [29,30]. Generative Adversarial Network (GAN) methods [31,32] have also been developed recently. However, all such approaches expose issues with the distortion metric that was used to train the networks [32]. Besides, DNN methods require significant training data and training computational effort.

Given the above, it is important to position our contribution—the SDMD method—as follows.

- We do not aim *quality wise* or *compression ratio wise* to compete with the compression effect of DNN techniques.
- We reduce significantly the blocking and banding artifacts of transform domain coding methods.
- We do not need any training data or expensive training procedures.
- We offer full control on how the compression works by the exposed free parameter of our method.
- Conceptually, we show that spline-based MATs are an efficient and effective tool for color image compression, which is, to our knowledge, the first result in this area.

## 3. SDMD Method

Our SDMD method (Figure 1) combines the advantages of CDMD (Section 2.1), which encodes *color* images with those of SMAT (Section 2.2), which compactly encodes MATs for *binary* images using B-splines. Moreover, besides simply integrating CDMD and SMAT, we propose three improvements that increase compression and quality: adaptively encoding upper vs. lower threshold-sets (Section 3.1); separately treating chrominance and luminance (Section 3.2); and removing Y-structures from the skeletons (Section 3.3).

### 3.1. Adaptive Layer Encoding

**Figure 2:**
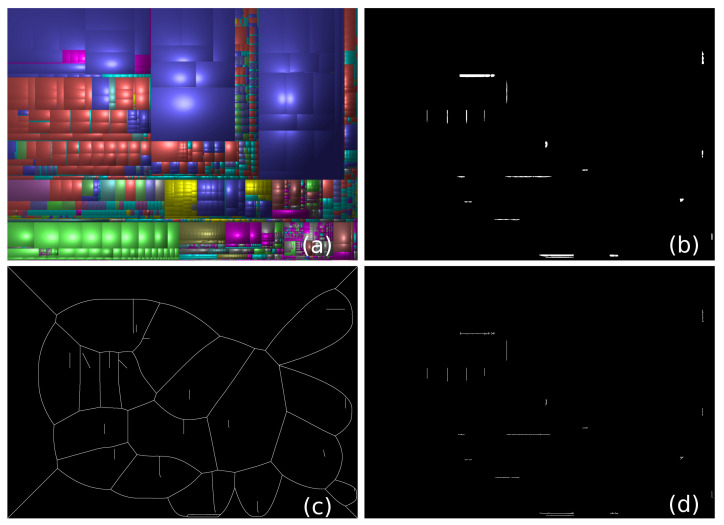
Adaptively encoding upper vs. lower threshold-sets. (**a**) Input image. (**b**) One threshold-set of the luminance channel in (**a**) for intensity *i* = 83. (**c**) The generated medial axis of $T_{83}^{↑}$. (**d**) The generated medial axis of $T_{↓}^{83}$, which is far less complex than, and thus preferred to, $T_{83}^{↑}$.

By definition, MATs require as input a *binary* image (Equations (1) and (2)). Hence, to encode a grayscale image *I* this way, we need first to decompose it in a set of threshold sets $T_{i}$. For this, CDMD proposed *upper* thresholding $T_{i}=\left\{x\in I\;|\;I(x)\geq i\right\},0\leq i<n$. Applying this thresholding for all selected layers *L* is however not good for compression. Figure 2a explores this by showing a cushion treemap image—a well-known visualization for hierarchical information [33]. Figure 2b shows one of its upper threshold sets $T_{i}$ (for i=83). Here, the black area is the region $T_{i}$ to be skeletonized. If we do so, the obtained skeleton $S_{T_{i}}$ is quite complicated (Figure 2c), meaning, it requires many B-spline control points to store via SMAT. However, if we chose instead to encode the white areas (regions in *Ioutside*$T_{i}$) from Figure 2b, i.e., if we use a *lower* thresholding for this layer $T_{i}=\left\{x\in I\;|\;I(x)\leq i\right\}$, the resulting skeletons $S_{T_{i}}$ will be significantly simpler, see Figure 2d, leading to fewer B-spline control points needed to encode them. Hence, instead of using upper thresholding for *all* selected layers, we *adaptively* encode upper or lower threshold-sets as follows. Let $T_{i}^{↑}=\left\{x\in R^{2}\;|\;I\left(x\right)\geq i\right\},0\leq i<n$ and $T_{↓}^{i}=\left\{x\in R^{2}\;|\;I\left(x\right)\leq i\right\},0\leq i<n$ be the *upper* and *lower* thresholding operation [34], respectively. We choose between the two to compute $T_{i}$ by greedy optimization, i.e.,

(4) $$T_{i}=\left\{\begin{array}{c} T_{↓}^{i},\;\;if\;\;N_{T_{↓}^{i}}<N_{T_{i}^{↑}}\\ T_{i}^{↑},\;\;otherwise \end{array}\right.$$

where $N_{T_{↓}^{i}}$ is the number of B-spline control points needed to encode $T_{↓}^{i}$, and similarly, $N_{T_{i}^{↑}}$ for $T_{↑}^{i}$. Simply put: We evaluate how expensive it is to encode $T_{↓}^{i}$ vs. $T_{i}^{↑}$ for each selected layer and choose the cheaper encoding of the two.

**Figure 3:**
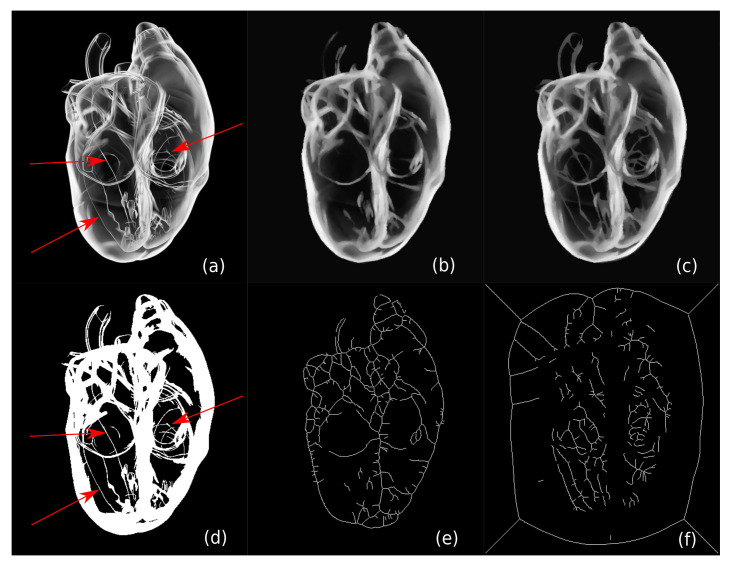
A comparison between reconstructions using the CDMD method (**b**) and our adaptive scheme (**c**) for a heart anatomy image (**a**). (**d**) The threshold-set (layer) 90 of (**a**). (**e**) The skeleton generated for $T_{90}^{↑}$. (**f**) The skeleton generated for $T_{↓}^{90}$.

Adaptively encoding upper vs. lower threshold-sets is a simple idea, but it can greatly improve the compression rate. Furthermore, it can even get better *quality*. Figure 3 shows an example. For a heart anatomy image (400×460 pixels, Figure 3a), images (b) and (c) are reconstructions by the CDMD method (using only upper threshold sets) and our new adaptive scheme, respectively. Compared with (c), (b) misses several thin curve structures in the image, which are marked with red arrows in (a). This can be explained on one of the selected layers $T_{90}$ (d). As said, CDMD only uses *upper* threshold-sets ($T_{90}^{↑}$) in which the shape to be encoded corresponds to the white areas in (d). Since the curves marked by the red arrows are one or two pixels thick, CDMD fails to generate skeletons for these curves (see image (e)), resulting in these curves missing in the reconstruction (b). In contrast, our adaptive method considers both $T_{90}^{↑}$ and $T_{↓}^{90}$. Since $N_{T_{↓}^{90}}$ obtained by (f) is less than $N_{T_{90}^{↑}}$, SDMD encodes $T_{↓}^{90}$, i.e., the black areas in (d). Since those black areas are thick enough to generate accurate skeletons, the white curves are preserved.

### 3.2. Per-Channel Encoding

**Figure 4:**
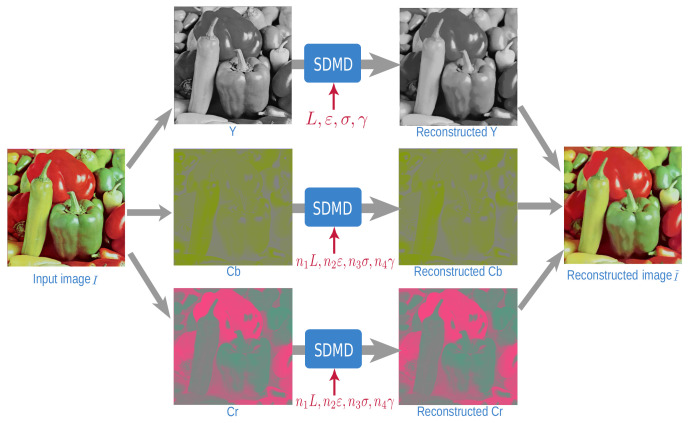
SDMD computation framework for color images. The red parameters indicate SDMD treats chrominance (Cb and Cr components) and luminance (Y component) separately.

Figure 1 shows the SDMD method only for a *grayscale* image. For color images, the CDMD method uses the RGB color space, handling each of the the channels *independently*, as in Figure 1. This has a high redundancy, preventing high compression. Later, saliency-based CDMD [35] improved this by using the YCbCr color space given that YCbCr can give better subjective quality than RGB due to its perceptual similarities to human vision [36,37]. However, this method used the *same* compression parameters for the three channels. We further take advantage of the human visual system’s lower acuity for chromatic differences (Cb and Cr components) than for achromatic difference (Y component) [38,39] to treat the three channels *separately*. We select fewer layers *L* for the two chrominance components (Cb and Cr) than for the luminance one (Y), and also compress Cb and Cr more than Y, using larger ε, σ, and/or γ. Concretely, given a user-selected parameter set (L,ε,σ,γ) for the Y-channel, we use the set $\left(n_{1}L,n_{2}\varepsilon ,n_{3}\sigma ,n_{4}\gamma \right)$ for the Cb and Cr channels. To find good values for $n_{1},\ldots ,n_{4}$, we fix three of the four coefficients to the value of one, in turn, and vary the fourth coefficient over its allowable range and evaluate the result. This led to $n_{1}=0.5,n_{2}=5,n_{3}=2,n_{4}=1$ as good values for producing high-quality results (see Figure 4).

**Figure 5:**
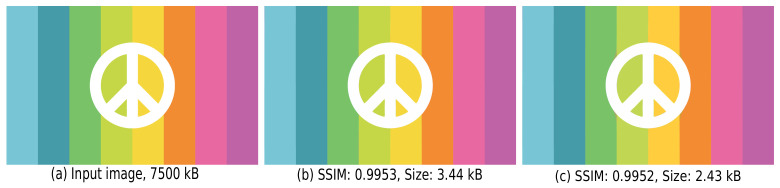
Benefits of compressing the three Y, Cb, and Cr channels separately. (**a**) Input image. (**b**) Using the same compression parameters for all three channels, i.e., *n*_1_ = 0.5, *n*_2_ = *n*_3_ = *n*_4_ = 1. (**c**) Using different parameters for the three channels, i.e., *n*_1_ = 0.5, *n*_2_ = 5, *n*_3_ = 2, *n*_4_ = 1 (see Figure 4).

Treating the three channels separately to compress chromatic components more than the luminance one allows greater overall compression without a significant effect on image quality. Figure 5 shows this by a sample image, in which (b) and (c) are the results when setting the *same* and *different* parameters for different channels, respectively. In this figure, SSIM is short for the Structural SIMilarity index [40], which measures how perceptually close $\tilde{I}$ is to *I*, where 1 indicates the two input images are identical, while 0 means the two are completely different. We see that using different parameters for different channels (Figure 5c) takes up about 30% less storage, while yielding results that are almost identical to (b) both visually and SSIM-wise.

### 3.3. Boundary Y-Structure Elimination

**Figure 6:**
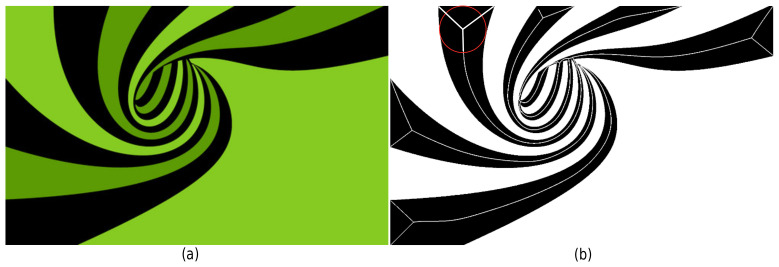
(**a**) Spiral shape image. (**b**) One of its threshold-sets $T_{128}^{↑}$ (black) and its corresponding skeleton $S_{T_{128}^{↑}}$ (white). A Y-like structure (bold white) and the medial circle (in red) of a Y-junction are also shown in (**b**).

**Figure 7:**
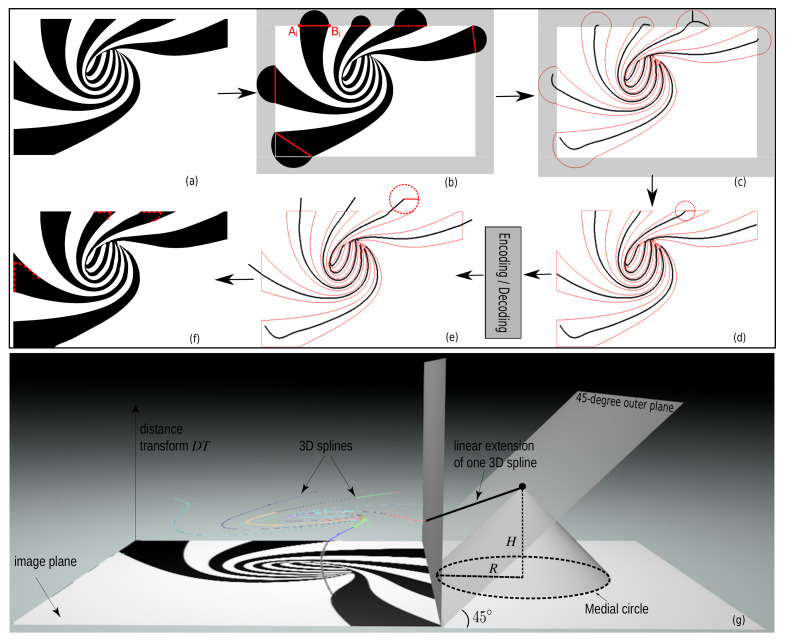
The pipeline of Y-structures elimination. (**a**) Layer $T_{128}^{↑}$ of the image in Figure 6. (**b**) Semi-disc extension of (**a**) with diameters in red. (**c**) The contour of (**b**) (in red) and the generated MATs (in black). (**d**) The cropped MATs of (**c**). A medial circle (in red) of an endpoint of the skeletons is also drawn in (**d**). (**e**) Extending MATs that touch the image boundary. A medial circle (in red) of an extended point is also shown in (**e**). (**f**) Reconstructed layer ${\tilde{T}}_{128}^{↑}$. Red dashed curves show the sharp corner results when directly using the cropped MATs in (**d**). (**g**) A schematic diagram of the extension (black line segment) of one border point of the 3D MAT curves. The 45-degree outer plane of the spiral image and the generated medial circle (black dotted line) of the extended endpoint are also shown in (**g**).

In contrast to Figure 1 and Figure 3, real-world images do not always have a background that *fully* surrounds the foreground image structures. Hence, their threshold-sets to be skeletonized will yield Y-like skeleton branches when the foreground structures reach the image boundary. Figure 6 shows this where the black (foreground) spirals reach the image boundary. The image is on purpose simple, for illustration aims. Encoding these Y-branches costs additional B-spline control points, thus lowering the compression rate. To get more compact skeletons, we present a Y-branch removal scheme, detailed next in Figure 7. For any layer $T_{i}$, let $\bar{A_{j}B_{j}}$ be the boundary segments corresponding to $T_{i}$ (black in Figure 6 and Figure 7) that touch the image boundary; see Figure 7b. Each such segment causes a Y-structure in the skeleton. To remove these Y-structures, we extend the size of the binary image $T_{i}$ to be skeletonized by adding a semi-disc to each boundary segment, centered at $(A_{j}+B_{j})/2$ and of radius $∥A_{j}-B_{j}∥/2$ (see Algorithm 1).

Due to this extension (Figure 7b), the computed skeletons (and their corresponding MATs) will reach out beyond the borders of the input image (Figure 7c). We next clip these MATs by the input image and fit the remaining structure with B-splines using SMAT (see Section 2.2). This gets rid of the unwanted Y-structures (see Figure 7d). When reconstructing these B-spline representations, we need to *prolong* the MATs where they touch the image border. If not, sharp corners, i.e., acute angles between the image boundary and the semi-disc diameter will be rounded-off, as shown by the red dashed curves in Figure 7f. This can be explained by the medial circle of an endpoint in Figure 7d. A bit larger medial circle of an *extended* point in Figure 7e, however, can totally cover the sharp corner. We extend the MATs by linear interpolation and then stop when the 3D MAT curves just reach the 45-degree outer planes of the image as this is where the generated medial circle is tangent to one of the image boundary edges, i.e., the medial circle is just about to leave the image, as shown in Figure 7g.
**Algorithm 1:** Semi-disc extension algorithm**Input**: Threshold-set $T_{i}$ **Output**: Extended $T_{i}$ to be skeletonized
1 Scan the pixel border of $T_{i}$ to detect the boundary segments $\bar{A_{j}B_{j}}$.
2 Enlarge $T_{i}$ by a band of thickness $\max_{j}∥A_{j}-B_{j}∥/2$ in all four directions.
3 Draw a semi-disc atop each segment $\bar{A_{j}B_{j}}$ with diameter $∥A_{j}-B_{i}∥$ and centered at $(A_{j}+B_{j})/2$.

**Figure 8:**
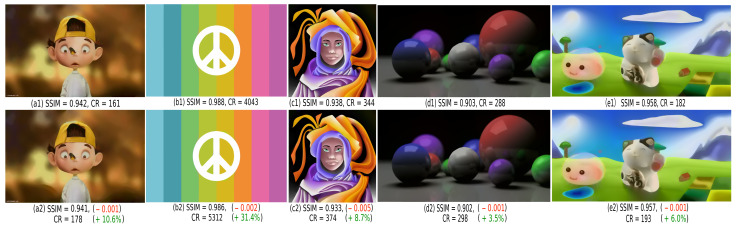
A comparison of the SDMD method for five images without Y-structures removal (**a1**–**e1**) and with this scheme used (**a2**–**e2**). For each image, we show the SSIM quality and compression ratio CR. We also indicate what we lost (in red) and what we gained (in green) in CR and SSIM when using the Y-removal design.

Figure 8 compares SDMD with (a2–e2) and without (a1–e1) Y-structure removal on five images that all have objects touching the image border. Additional examples also using more parameter settings are available in the supplementary material [41]. For each image, we also list its SSIM value (similarity to the uncompressed original, see Section 3.2) and its compression ratio CR, defined as the size of the original image divided by the size of the SDMD encoding (for details, see Section 4.1). Green and red numbers in Figure 8 indicate better and worse, respectively, SSIM and CR values for the Y-removal scheme as compared to using plain SDMD. From these values, we see that the Y-removal scheme increases the CR (from 3.5% to 31.4%), with negligible quality loss (around 0.002 SSIM decrease). The CR gain depends on how many objects in the image touch its borders. For instance, image (d) has only one object—the right green sphere—touching a small part of the border, so the Y-removal scheme boosts CR by only 3.5%. In contrast, image (b) has 8 color bands touching the image border along its entire extent, so the Y-removal scheme boosts CR by 31.4%.

## 4. Results

We comprehensively evaluate the proposed SDMD method from various angles, as follows.

- First, we build an evaluation benchmark (Section 4.1);
- We study how SDMD depends on its free parameters (Section 4.2);
- We quantitatively assess the adaptive layer and per-channel encoding extensions proposed earlier (Section 4.3);
- We compare our method with the original CDMD method, the well-known JPEG technique, and the recently developed JPEG 2000 and BPG. (Section 4.4);
- Finally, we show how SDMD performs on images of different resolutions (Section 4.5).

### 4.1. Benchmark

The SDMD encoding consists of a tuple $\left(w,h,\left\{l_{i}\right\}\right)$, i.e., the width *w* and height *h*, in pixels, of the input image *I*, and the *L* selected layers $l_{i}$. Each layer $l_{i}=\left(i,f,\left\{b_{i}^{k}\right\}\right)$ encodes the layer number or intensity value *i*, a flag bit *f* that tells whether this layer needs to be flipped or not (see Section 3.1) and a set of B-splines $\left\{b_{i}^{k}\right\}$ encoding the layer’s MAT. Each B-spline $b_{i}^{k}=\left(d_{i}^{k},\left\{c_{j}\right\}\right)$ consists of a degree $d_{i}^{k}\in N$ and a set of control points $c_{j}\in R^{3}$ (see Section 2.2).

SDMD is evaluated based on two factors: **Quality** *Q* of the reconstruction $\tilde{I}$ of the input image *I*, which is measured by the SSIM difference of the two images (see Section 3.2) and the **Compression ratio** CR, defined as $CR=|I|/|SDMD(\tilde{I})|$, i.e., the byte-size of the original image *I* divided by the byte-size of the SDMD encoding of $\tilde{I}$, which has been described above.

To evaluate the SDMD method comprehensively, we need to create a benchmark involving multiple image types. Indeed, as earlier work using CDMD to represent images has shown [3,35], MAT-based image representations work best for images consisting of relatively large shapes overlaid on a smooth background. This is not surprising given that MATs were also originally found to be most effective for the analysis (and representation) of *shapes* [7]. As such, we also target our method to represent color imagery of a similar type. We found several classes of imagery that fall within this typology, namely scientific visualizations of continuous data (scalar and vector fields), medical images (from, e.g., CT, X-ray, and MRI scans), synthesized images using graphics rendering and vectorization methods [42], graphics art (logos, graphics design), and cartoon images. High-quality, low-size representations of these image types are needed for many applications such as remote browsing of specialized content (SciVis, medical) or general-purpose content (webpages) when, e.g., using low-speed connections. For our evaluation, we consider a benchmark with the above-mentioned five image types, each type having at least 10 images. Table 1 shows a summary of the benchmark.

### 4.2. Parameters Effect

As Figure 1 shows, SDMD depends on four parameters: the number of selected layers *L*, the size of removed islands ε, the saliency threshold σ, and the spline fitting tolerance γ. To find a good trade-off between *Q* and CR, we fix, in turn, three of the four free parameters *L*, ε, σ, and γ to empirically-determined values and vary the fourth parameter over its allowable range via uniform sampling. This method is also applied in [3] and [35] and is much simpler and faster than the usual hyper-parameter grid-search used, e.g., in machine learning [43].

**Figure 9:**
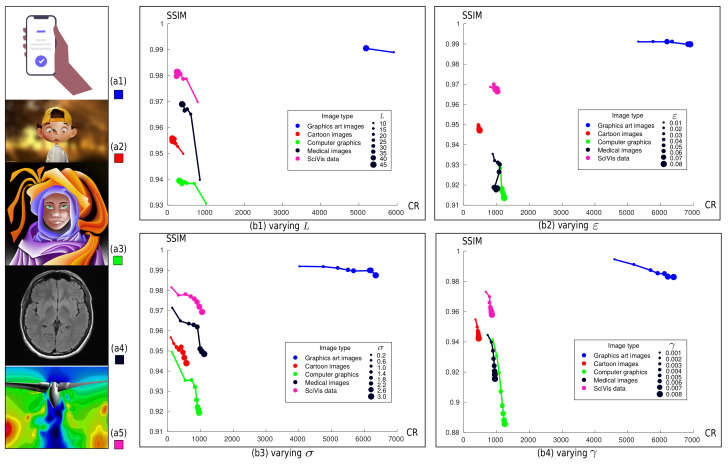
Trade-off between *Q* (SSIM) and CR for five image types (**a1**–**a5**) as a function of parameters *L* (**b1**), *ε* (**b2**), *σ* (**b3**), and *γ* (**b4**), respectively. The box colors in (**a1**–**a5**) corresponds to the five colors of scatterplots in (**b1**–**b4**).

Figure 9 shows the results of this parameter search for five images, one of each type in the benchmark. The actual images are shown to the left. The subsequent four plots (b1–b4) show how *Q* and CR are related when varying each of the *L*, ϵ, σ, and γ parameters, while keeping the other three fixed to their default values. The colored line plots indicate the *Q* vs. CR graphs for each image, with dot sizes along these lines indicating the varying parameter’s values (see the legends). Overall, the plots in Figure 9b1–b4 show a negative correlation between CR and Q for all images and parameter variation experiments, which is expected. Indeed, higher quality leads to a lower compression ratio.

Figure 9b1 shows the trade-off between *Q* and CR as a function of the number of layers *L*. We sample *L* from 10 to 45 with a step of 5, following observations in [3] stating that *Q* and CR hardly change for *L* > 40. This is also visible from (b1): when the number of layers *L* increases to around 40, the points along a line almost overlap. This is most salient for the blue curve (graphics art images), where *Q* and CR do not change at all when L>10. In addition to *L*, the other three parameters are set to ε=0.01, σ=1.0, and γ=0.002. Chart (b1) also shows that except graphics art images (a1), the other four curves have a ‘tail’ pointing downward, indicating a notable drop of SSIM for low *L* values for a minimal increase in CR. As such, we deem that a value *L* of 10 to 15 for graphics art images and 15 to 20 for the other four types are good preset values. Figure 9b2–b4 show quite similar trends for σ, γ, and ε, as discussed above for *L*. Lower parameter values yield lower CR and higher SSIM, and conversely.

Given all the above, we settle to the preset values (or ranges) L∈[10,20], ε=0.01, σ∈[0.6,1.4] and γ=0.002 that give a good SSIM vs. CR tradeoff. We next use SDMD with parameters in these ranges to evaluate the method on more images and also compare its results with other compression methods.

### 4.3. Quantitative Evaluation of Adaptive Layer and Per-Channel Encoding

Section 3 details three improvements to the original CDMD method: adaptively encoding upper or lower level-sets, separately treating chrominance and achrominance channels, and eliminating Y-terminations in the MAT. We have discussed the added value of Y-termination removal already in Section 3.3, showing that it produces a significant CR boost for basically no SSIM decrease. As such, we next focus on the evaluation of the adaptive layer and per-channel encoding schemes.

**Figure 10:**
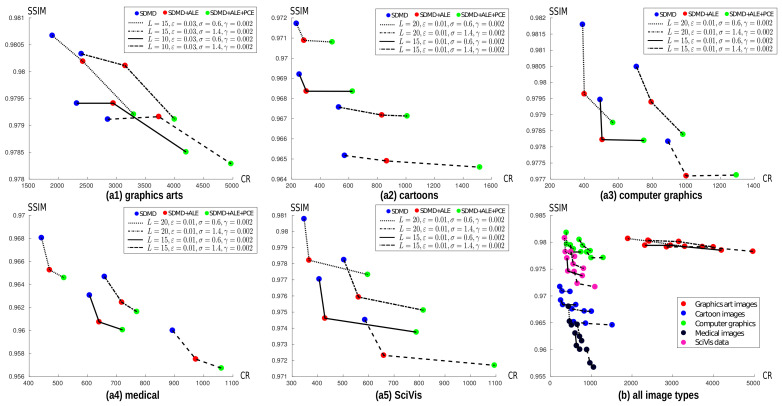
The average SSIM vs. CR for basic SDMD (blue dots), SDMD with adaptive layer encoding (ALE, red dots) and SDMD with both an adaptive layer and per-channel encoding (ALE + PCE, green dots) for graphics art images (**a1**), cartoon images (**a2**), computer graphics (**a3**), medical images (**a4**), and SciVis images (**a5**). (**b**) Summarization of the first five plots, with colors indicating image types.

Figure 10 shows the average SSIM vs. CR for our five image types for three SDMD schemes, i.e., the basic SDMD method (blue dots), SDMD with adaptive layer encoding on (red dots), and SDMD with both adaptive layer encoding and per-channel encoding on (green dots). Each of the five charts corresponds to one image type. Each polyline in a chart corresponds to a different parameter setting, as indicated in the legend, following the parameter-setting discussion in Section 4.2. Finally, each colored dot in a polyline corresponds to one of the three SDMD schemes mentioned above. For graphics art images, as Figure 9 showed, fewer layers *L* and slightly larger island thresholds ε can produce good results, so we chose for these the parameter combination L∈{10,15},ε=0.03,σ∈{0.6,1.4},γ=0.002. For all the other image types, more layers *L* and a slightly smaller ε are used, as indicated in the legend in Figure 10.

For ease of reading, Table 2 aggregates the results detailed in Figure 10, showing the loss (↓) and gain (↑) in SSIM and CR, respectively, when using the adaptive layer encoding (ALE) and ALE plus the per-channel encoding (PCE). From this table and Figure 10, we see that the quality loss is very little (from 0.0002 to 0.0032) for all image types, regardless of whether we use only ALE or both ALE and PCE. We also see that, for medical imagery, the gain in CR of both ALE and PCE is the smallest, 18% on average. This is mainly because most such images are grayscale. Hence, the effect of per-channel encoding (PCE) is almost zero. In contrast, for cartoon images, ALE + PCE yields an increase of CR of 128%, that is, the two enhancements more than double the compression ratio as compared to plain SDMD. This can be explained by the fact that most cartoon characters have a thin black outline. When lower thresholding such images, we obtain threshold sets that have very thin components, similar to the one shown in Figure 2b. Hence, as in that example, ALE will greatly simplify the MATs to be encoded for cartoon images, yielding higher CR values.

Finally, Figure 10b shows all the results from the previous five charts in the same figure but in a single image. Note that the ranges of both the CR and SSIM axes of all charts are different, chosen so that we ‘zoom in’ in each case on the range in which the actual data varies. Figure 10b lets us compare how SDMD (with the ALE and PCE adaptations) performs across different image types. We see here, in more detail than Table 2, that SDMD works particularly well for graphics art images (red dots). We also see that the ALE and PCE adaptations yield only small CR gains for the medical images (black dots are distributed along almost vertical lines). For the cartoon images, scientific visualizations, and computer graphics images, the two adaptations perform in-between medical images and graphics arts images, that is, increase CR for a limited SSIM decrease. Given these results, we conclude that both adaptations are of added value, as they create a negligible SSIM loss for a significant increase in CR for all image types and all parameter combinations.

### 4.4. Comparison with CDMD and JPEG

In this section, we compare the improved SDMD method—using the adaptive layer and per-channel encoding which showed added-value in the evaluation in Section 4.3—with the CDMD method and JPEG for all our benchmark images.

#### 4.4.1. Comparison with the Original CDMD Method

**Figure 11:**
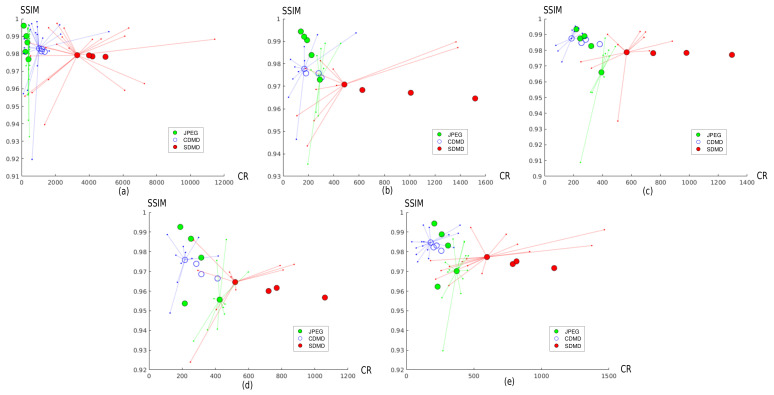
Comparison of CDMD (blue dots), JPEG (green dots), and SDMD (red dots) for graphics art images (**a**), cartoon images (**b**), computer graphics (**c**), medical images (**d**), and SciVis data (**e**). The actual image data (smaller dots) are connected to the corresponding average value (larger dots) for one parameter setting of each method.

Figure 11 compares the results of SDMD (red dots) and the original CDMD method (blue dots) under the same parameter settings. Each plot in the figure represents images of a different type. Similar to SDMD, we define compression ratio (CR) as $CR=|I|/|MAT(\tilde{I})|$, where MAT is the size (in bytes) needed to store $S_{T_{i}}$ with the delta-encoding scheme proposed by CDMD, rather than the B-spline scheme used by SDMD. The large dots in the plot show the CR and SSIM averages over all the benchmark images for one parameter setting. Hence, different large dots correspond to different parameter settings. To show more details, we also display a star plot for one of the parameter settings, i.e., connect the large dot (average over all images) with small dots that indicate the CR and SSIM values for every individual image. Hence, small stars indicate little deviation in CR and SSIM from the average over the image benchmark; large stars indicate more variability of these metrics as a function of the actual image.

Figure 11 shows that the star plot shapes of CDMD and SDMD are quite similar. In other words, CDMD and SDMD exhibit a similar dependency on the image type. This is due to the fact that SDMD inherits the thresholding and skeletonization method of CDMD. More importantly, the points plotted for SDMD (red) are always at the bottom right of those of CDMD. That is, SDMD always gets a significantly higher CR for only a small decrease in quality. Quantitatively, on average, compared with CDMD, SDMD reduces SSIM by 0.003 (art graphics images (a)), 0.008 (cartoon images (b)), 0.008 (computer graphics images (c)), 0.01 (medical images (d)), and 0.008 (SciVis images (e)). On average, compared to CDMD, SDMD increases compression by a factor of 3.4 (a), 3.7 (b), 3.2 (c), 2.5 (d), and 3.8 (e), which we deem to be a very substantial improvement.

#### 4.4.2. Comparison with JPEG

Figure 11 also allows comparing SDMD with JPEG, the latter run under five quality settings, i.e., 10%, 30%, 50%, 70%, and 90%. For each such quality setting, we plot the average CR and SSIM of JPEG as a single green dot in each chart in Figure 11. Green dots are sorted right-to-left by increasing quality setting values—that is, the higher the JPEG quality setting, the lower the obtained CR. If we compare SDMD with JPEG, we see that SDMD cannot reach the same SSIM values as when JPEG uses its 90% quality setting—the topmost green dots in each plot are above the topmost red dots. However, the difference in quality (SSIM) is quite small, if we look at the vertical spread of the green vs. red dots—about 2% on average. Separately, we see that SDMD always gets higher compression rates than JPEG for *all* situations—red dots are always (significantly) to the right of the green dots. We also see that the green dots are spread far more along the vertical (SSIM) axis than the horizontal one, indicating that JPEG’s quality setting can influence SSIM far more than CR. In contrast, the red dots are spread far more along the horizontal (CR) axis than the vertical ones, indicating that SDMD’s settings can influence compression significantly for only a small drop of quality. In particular, if we are after strong compression, SDMD performs better than JPEG: Compared to JPEG with a quality of 10% (the rightmost green dot in each plot), SDMD always gets both higher CR and better quality, except for the cartoon images. When fewer layers and larger saliency thresholds are used (rightmost red point in each plot), SDMD not only gets better quality but yields a compression that is 12 (a), 3.3 (c), 2.5 (d), and 2.9 (e) times higher than that of JPEG with a quality setting of 10%.

**Figure 12:**
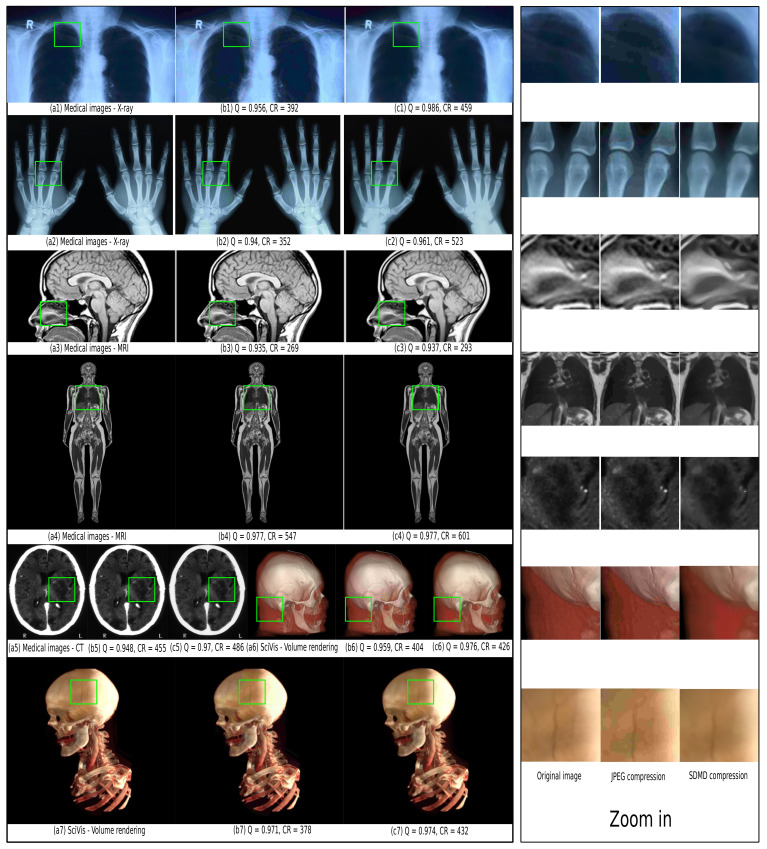
Left pane: Comparison of JPEG for quality set to 10% (**b1**–**b7**) with SDMD (**c1**–**c7**) for 7 input images (**a1**–**a7**) of medical type. For each result, we show the SSIM quality Q and the compression ratio CR. Right pane: Zoomed-in areas, marked in green in the images in the left pane, show subtle differences between the original, JPEG, and SDMD.

**Figure 13:**
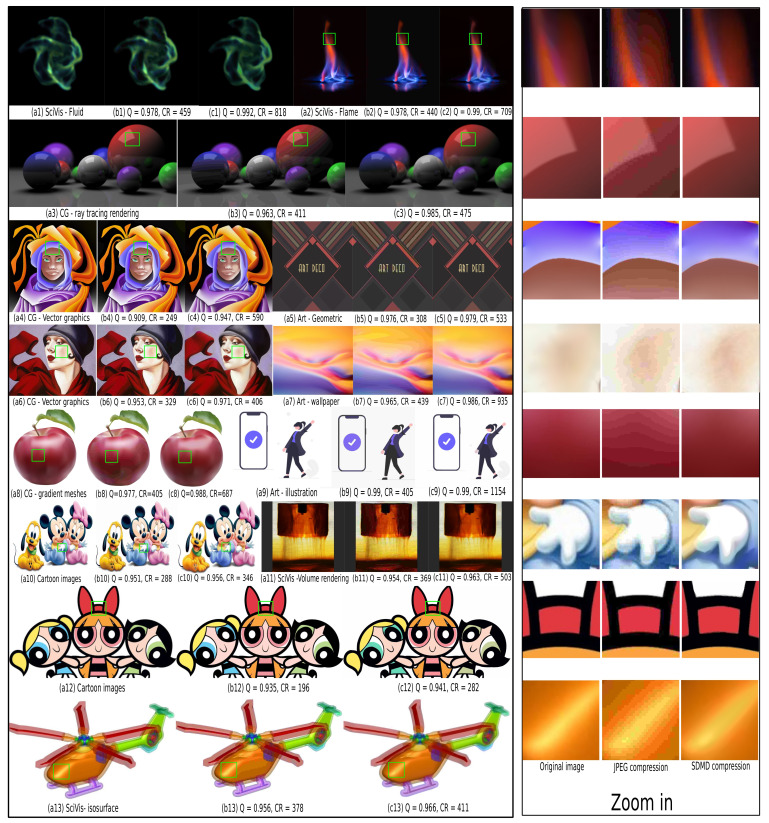
Left pane: Comparison of JPEG for quality set 10% (**b1**–**b13**) with SDMD (**c1**–**c13**) for 13 input images (**a1**–**a13**) which span four types in Table 1. For each result, we show the SSIM quality Q and compression ratio CR. Right pane: For each row, we selected an area of one image to zoom in for detailed comparison.

Figure 12 and Figure 13 further refine the above insights by showing 20 images, spanning the five types of our benchmark (Table 1), compressed by JPEG (with a quality of 10%) and with SDMD. The results for the entire benchmark are available in the supplementary material [41]. From the zoomed-in areas of specific blocks on the right, we observe that JPEG with a low-quality setting generates obvious artifacts such as checkerboarding (Figure 12b1,b7), banding (Figure 13b2–b4,b8,b13), background color changing (Figure 13b2,b9), and object details missing (Figure 13b9). In contrast, SDMD yields better quality (SSIM) and does not exhibit the aforementioned artifacts, leading to images which, we argue, are almost indistinguishable from the originals. Separately, SDMD also achieves much higher compression rates than JPEG, especially for the scientific visualization (Figure 13c1,c2), vector graphics (Figure 13c4), abstract shapes (Figure 13c7), and illustration (Figure 13c9) image types. The good performance of SDMD on medical images (Figure 12c1–c5) suggests that SDMD could be very well suited and superior to JPEG in the context of remote/online viewing of medical image databases.

#### 4.4.3. Additional Comparisons

As stated in Section 2.3, tens of image compression methods exist. We did not perform an evaluation against these since, as already outlined in Section 1, our main research question was to explore the potential of spline-based MATs as an alternative tool to image *representation*, which includes image compression applications (Section 4) but also other applications such as super-resolution images generating (Section 5.1) and salient feature-preserving simplification (Section 5.2). Therefore, for image compression, we only evaluated SDMD in Section 4 against the arguably most widely used compression method, i.e., JPEG. Given the positive results outlined by the comparison with plain JPEG, next, we explored how SDMD compares with newer variants proposed in the literature as replacements for JPEG that increase compression ratios while preserving image quality, i.e., BPG [44] and JPEG 2000 [23].

**Figure 14:**
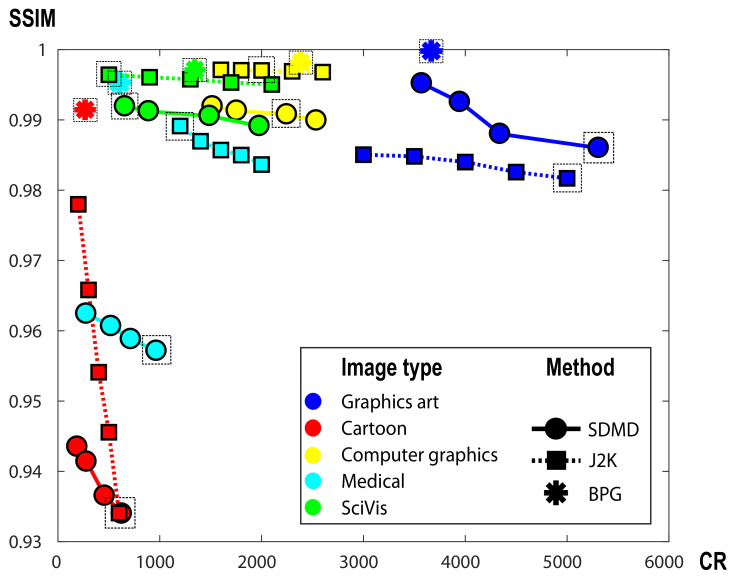
A comparison of SDMD (dots), JPEG 2000 (squares), and BPG (stars) for a graphics art image (blue), a cartoon image (red), a computer graphic (yellow), a medical image (cyan), and a SciVis image (green).

**Figure 15:**
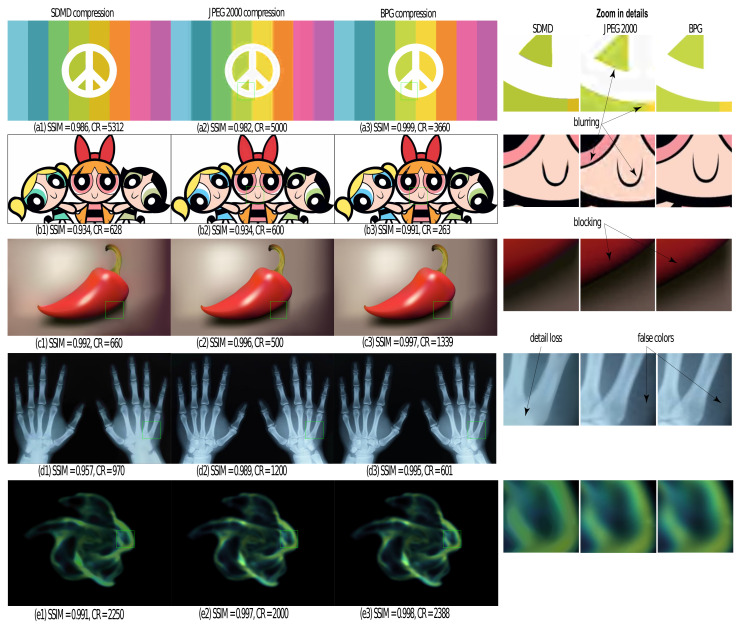
A comparison of SDMD (**a1–e1**) with JPEG 2000 (**a2–e2**) and BPG (**a3–e3**) for 5 input images, one of each type in Table 1. For each result, we show the SSIM quality Q and compression ratio CR. The right three columns show selected areas zoomed in on the three images in the same row to the left, for detailed comparison.

Figure 14 compares SDMD, BPG, and JPEG 2000 for five images, one of each type in our benchmark. The actual images are shown in Figure 15. For each image, we run SDMD (solid line) under four parameter settings, JPEG 2000 (dotted line) under five compression settings, and BPG (star markers) under its default setting. As visible, for the graphics art image (blue dots), SDMD produces both higher quality and compression than JPEG 2000. For the other four types, JPEG 2000 generates better quality and/or higher CR than ours. For all these five types, BPG generates higher SSIM than SDMD when the CRs of the two are similar. However, for all above cases, the differences, both in Q and CR, are quite small.

Figure 15 refines this insight by showing the reconstruction results under the quality setting indicated in Figure 14 with the dashed box. Overall, the three methods perform visually very similarly, as already indicated by the similar SSIM values in Figure 14. The zoomed-in areas show a few subtle differences: For strong-contrast images, such as the first two in Figure 15, JPEG 2000 tends to create some small-scale blur artifacts. This is also seen in the fact that, for the first image in Figure 15, SDMD yields both higher SSIM and CR than JPEG 2000. Compared to BPG, SDMD’s results are very similar. For the third image, which exhibits a smooth luminance gradient in the shadow area, SDMD captures this gradient quite well. In contrast, JPEG 2000 and BPG cause a slight amount of blocking artifacts. For the fourth image, JPEG 2000 and BPG create a small amount of blocking and false colors (purple) in the near-constant-luminance, dark blue, area. In contrast, SDMD does not have such problems but suffers from loss of small-scale, faint, details—due to its selection of threshold-sets to be encoded (Section 4.2). Finally, for the fifth image, all methods produce basically visually identical results.

Summarizing the above observations, we conclude that SDMD can create images that are visually very similar to those produced by modern variants of JPEG, with a slight loss in quality and compression ratio.

### 4.5. SDMD Performance on Images of Different Resolutions

All images in our benchmark have quite high resolutions ($2000^{2}$ to $3000^{2}$ pixels). We next test how SDMD performs on images of different resolutions. For this, we start with a high-resolution image and generate *m* downscaled images from it using ImageMagick [45]. Next, we run SDMD on the total m+1 images and study how SSIM and CR vary as a function of the image size.

**Figure 16:**
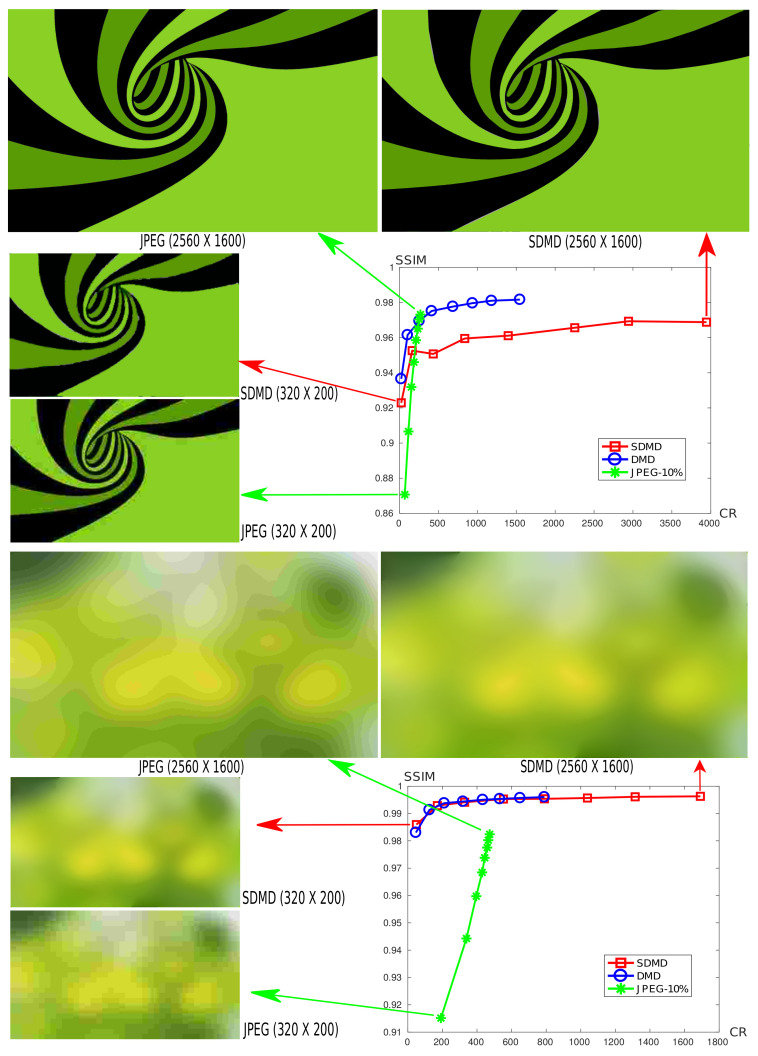
A comparison of SSIM vs. CR for the SDMD (red), CDMD (blue), and JPEG (green) methods for two graphics art images of 8 different resolutions each, from 320 × 200 to 2560 × 1600 pixels. Note that the image sizes shown in the figure are not proportional to their actual sizes, for space reasons.

Figure 16 shows this analysis for two graphics art images of m=8 different resolutions each, from 320×200 to 2560×1600 pixels. For additional insights, we also compare SDMD with CDMD and JPEG on these images. The charts show the CR vs. SSIM plots as we vary the image resolution. That is, for a given method, we plot a polyline of m=8 points, indicating the respective CR and SSIM values for all the resolutions. We also show the actual images for the lowest and highest, respectively, resolutions for both SDMD and JPEG.

Several insights can be obtained as follows. First, we see that SDMD dominates CDMD in CR values, with no quality loss whatsoever (green background image) and a minimal quality loss of about 2% (spiral shape image). We also see that quality increases with input image size. For example, for the spiral shape image at the lowest resolution (320×200 pixels), both JPEG and SDMD yield a quite low quality, with SDMD being about 5% better than JPEG. The loss of quality is also visible in the actual image snapshots (shown on the left of the chart) that exhibit fuzzy effects. However, the reasons for fuzziness are different: For JPEG, this is caused by blocking artifacts; for SDMD, the fuzziness is caused by the inaccurate reconstruction of threshold-sets due to the spline fitting error. Still, the SDMD reconstruction looks overall smoother and sharper, as also reflected by its higher SSIM score. For the largest resolution image (2500×1600 pixels), both JPEG and SDMD produce visually good reconstructions and have similar (high) SSIM scores. However, SDMD compresses about 16 times more than JPEG. Interestingly, for the second image example (green background image), SDMD produces a quite smooth reconstruction both at the minimal and maximal resolution. In contrast, JPEG shows a pixelated reconstruction for the lowest resolution and strong banding artifacts for the highest resolution. Here, again, SDMD compresses better than JPEG: about 4 times more for the highest resolution.

**Figure 17:**
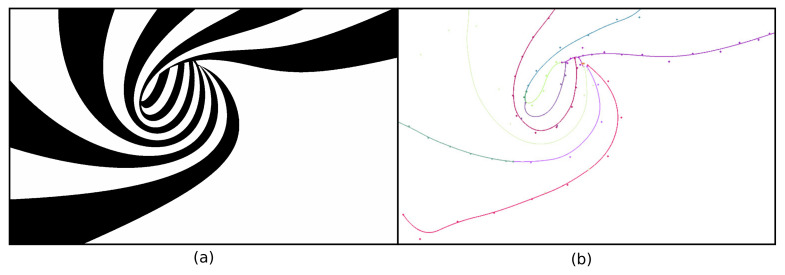
One of the level-sets for the geometric image in Figure 16 (**a**) and its corresponding B-spline MAT representation (**b**).

From Figure 16, we also observe that $S_{SDMD}>S_{CDMD}>S_{JPEG}$, where $S_{SDMD}$ indicates the *slope* of the curve of SDMD, and similar for CDMD and JPEG. This means that as the input image becomes larger, the compression rate of the SDMD method increases the fastest, followed by CDMD, and finally by JPEG. The reason for this is determined by the compression principle of the three methods. JPEG compresses images by splitting them into 8×8 blocks; CDMD captures shapes in the image using MATs; and SDMD further encodes skeletons with B-splines. Intuitively, we can say that the ‘compression unit’ is two-dimensional (block) for JPEG, one-dimensional (skeleton branch pixel-chain) for CDMD, and zero-dimensional (B-spline control point) for SDMD, respectively. Figure 17 further illustrates this by showing (a) one of the threshold-sets for the spiral image in Figure 16 and (b) its corresponding spline representation. As already explained, SDMD only stores the locations of the *control points* shown in Figure 17b. Hence, if we uniformly scale the input image by any arbitrary factor, the control points will stay the same in terms of relative positions and number, or only change very little due to small-scale sampling issues related to the fixed pixel-grid. Hence, SDMD will compress a larger version of the spiral image as efficiently as a smaller version.

## 5. Applications

Besides image compression, SDMD provides ways to create super-resolution images and selective encoding of salient features, as discussed next in Section 5.1 and Section 5.2, respectively.

### 5.1. Super-Resolution Images

**Figure 18:**
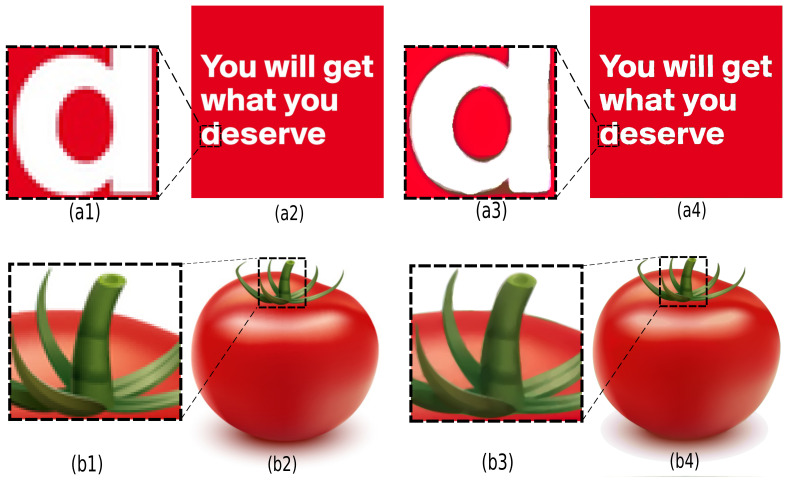
The super-resolution effect on a text image (**a**) and a graphic generated by gradient meshes (**b**). (**a1,b1**) Enlarged areas of the input images (500^2^ pixels) (**a2,b2**). (**a3,b3**) Enlarged areas of SDMD reconstructions (3000^2^ pixels) (**a4,b4**).

Image super-resolution (SR) is a popular technique for constructing higher-resolution images from low-resolution ones. Recently, AI researchers have used powerful deep learning algorithms for SR tasks and achieved high quality [46,47,48]. However, as pointed out recently in [49], deviations in the characteristics of the training data and test images can cause significant performance degradations. Besides, such approaches also require considerable training data. In comparison to deep-learning methods, SDMD is fully generic and does not need training data or special interpolation tricks [50]. To perform SR, SDMD simply *rasterizes* the reconstructed splines at the desired target resolution during step 4 in Figure 1. As this occurs during reconstruction, *generating the SR result does not incur any extra storage*. Figure 18a2,b2 show a text image and a graphic generated by gradient meshes, both at a relatively low 500×500 pixels resolution. Images (a4) and (b4) show the SDMD reconstructions of these two images at a six-times higher resolution, i.e., 3000×3000 pixels. Any other target resolution can be used directly given a computed SDMD encoding of an image. As seen from the enlarged areas in Figure 18, the SR reconstruction improves the discretization artifacts of the original images while keeping the reconstructed boundary clear and smooth.

### 5.2. Salient Detail Encoding

**Figure 19:**
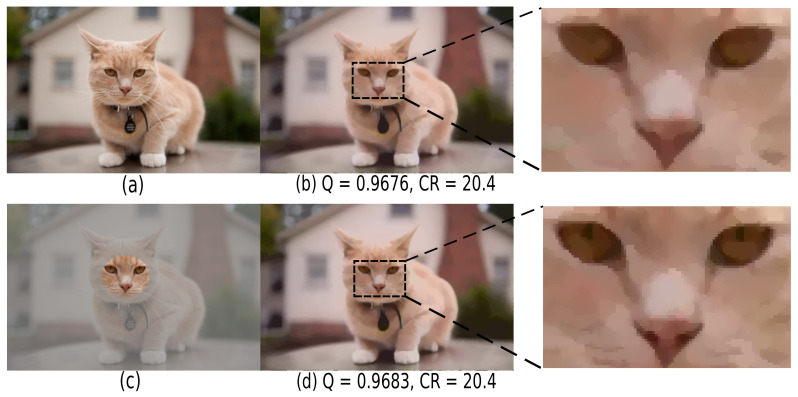
The benefits of handling salient information. (**a**) Input image. (512 × 337) (**b**) The SDMD reconstruction with the enlarged area of the face on the right. (**c**) The manually set salient area. (**d**) The SDMD reconstruction considering salient information.

As explained in Section 2.1, SDMD simplifies an image *globally*, e.g., removing islands smaller than a *global* threshold ε or pruning skeletal branches with a saliency below a *global* threshold σ. This is not desirable in practice for certain images that contain different levels of detail. Figure 19 gives an example. For the input image (a), (b) shows the SDMD reconstruction using the default global island threshold ε=0.001. As visible from the enlarged area on the right, SDMD loses some small but important details of the cat’s face. Further reducing ε can alleviate this, but this also allocates more information to encode the (less important) background, thereby reducing compression. To address this, we allow users to define salient areas based on manually drawn maps, as shown in Figure 19c. Based on these maps, we use a low threshold for salient areas (ε=0.0005 in this example) and a larger threshold for regions outside the important areas (ε=0.0015 in this example). This way, we obtain an identical CR as when using the global ε setting. However, the quality slightly increases since we now preserve more details in the salient area (d). Apart from manually designed maps, automatically computed saliency-maps generated by supervised methods [51] or unsupervised methods [52,53,54] can also be used out-of-the-box with SDMD.

## 6. Discussion

We next discuss several aspects of our SDMD image compression method.

**Speed:** SDMD is linear in the number of pixels of the input image. To gain more insights, we measured the time SDMD needed for a color image at eight different resolutions on a Linux PC with an Nvidia RTX 2060 GPU. Table 3 lists the timings of the four key steps of SDMD (skeletonization, spline fitting, reconstruction, and interpolation). Each step shows the time needed to process all three channels (YCbCr). Skeletonization and reconstruction are relatively less expensive operations as they are very efficiently implemented on the GPU. Interpolation is a bit more expensive since it needs to compute distance transforms for all the selected layers and use them to perform a per-pixel interpolation (Section 2.1). Spline fitting executes the least-squares optimization and the adaptive-degree fit-and-split algorithm in [4], which dominates the running time.

**Ease of use:** SDMD has four free parameters that affect the trade-off between the compression ratio and image quality, as discussed in detail in Section 4.2. The meaning of these parameters is quite straightforward: *L* determines how many layers (image intensities for a grayscale image) are used for the reconstruction; ε controls the maximum size of small-scale details that are removed; σ controls how much to smooth isophote or isochrome contours in an image; and γ tells how accurately B-splines fit the MAT, i.e., how precisely we want to encode the position and shape of objects in an image. More importantly, Section 4.2 provides good defaults for all these parameters and also shows that the method is predictable and robust when these are varied away from their presets.

**Replicability**: We implemented the entire SDMD method in C++. We compute MAT and reconstruct the threshold-sets from a rasterized spline using the public CUDA code provided at [55]. We provide the full source code of SDMD, as well as the image benchmark used in this paper, for replication purposes [41].

**Figure 20:**
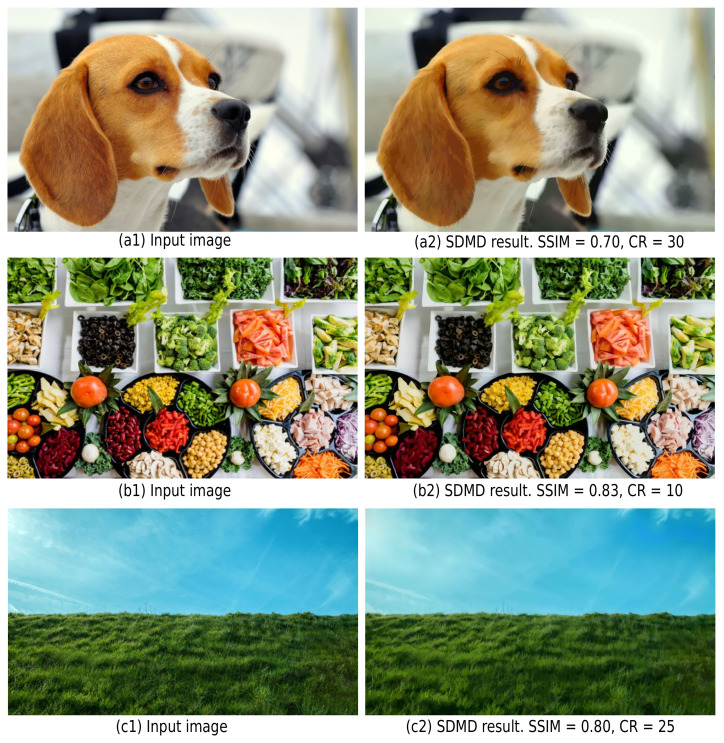
Poor performance (both in SSIM and CR) for SDMD when dealing with images with many fine details, such as animal furs (**a**), trivial objects (**b**), and greenery (**c**). The sizes of the three input images are all 2560 × 1600.

**Limitations:** While SDMD can handle any image type and resolution, it exhibits limited performance for small images (see Figure 16). Figure 3 shows an additional result in this sense for an image of $400^{2}$ pixels. Furthermore, SDMD cannot get better compression *and* quality than JPEG for all image types even for large resolution images: Like CDMD, SDMD is not good at handling images with many fine details (high spatial frequencies). Figure 20 illustrates this by showing three such images. Their SSIM scores are quite low due to the fact that SDMD cannot encode and reconstruct very thin image details. Additional insights shown in Section 4.4.3 show a similar positioning of SDMD vs. more modern variants of JPEG, specifically BPG and JPEG 2000. However, we argue that, for any practical purpose, the SDMD representations actually look visually very similar to the input images and are largely free of obvious artifacts, such as color banding, checkerboarding, or false hues. This may suggest that the SSIM metric used to compare images is too strongly penalizing such small details and opens the broader question on which metrics should be further considered to compare lossy-compressed images *in practice*. This is an important question that, albeit out of our current scope, deserves further research.

## 7. Conclusions

We have presented SDMD, a method for compressing color and grayscale images by encoding dense medial descriptors obtained from the images’ threshold sets with accurate B-splines. SDMD adapts the existing CDMD method—proposed for encoding images with medial descriptors— in four directions, namely (a) replacing the expensive pixel-chain coding of medial descriptors by B-splines, (b) adaptively encoding upper or lower threshold-sets to minimize the amount of storage space, (c) separately treating chrominance and achrominance, and (d) eliminating medial Y-structures that touch the image boundary. To study the effectiveness of our method, we considered a benchmark of five different image types, each type having at least 10 images. The quantitative evaluation showed that our adaptations of CDMD greatly improve compression at only a small quality loss. Furthermore, the proposed SDMD delivers superior compression to the well-known JPEG method at similar or even better quality, especially for large images. Finally, we show how SDMD can be used out-of-the-box to generate super-resolution images and also can be adapted to perform local salience-based compression. SDMD is implemented on the GPU, making its application take only a few seconds on a modern PC for images up to $2000^{2}$ pixels.

Several future work directions are possible. First, more extensive evaluations are of added value, considering more compression methods, e.g., deep neural network approaches. Secondly, extending SDMD beyond grayscale or color image representations to encode 2D and 3D scalar fields for scientific visualization is an interesting avenue to follow. Finally, we aim to explore the potential of dense medial descriptors for more applications, e.g., salient corner detection in general images.

## Figures and Tables

**Table 1:** The benchmark of five image types (available at [41]) used throughout this work for evaluating SDMD.

Type	Description	Quantity
SciVis data	Scientific visualizations (scalar and vector fields)	15
Medical images	Images generated by CT, X-ray and MRI scans	10
Computer graphics	Images generated by rendering and vectorization	10
Graphics art images	Simple shapes such as clip art, logos, and graphics design	20
Cartoon images	Animated cartoons and comic strips	10

**Table 2:** Decrease in SSIM (↓) and increase in CR (↑) for the adaptive layer encoding (ALE) and ALE plus the per-channel encoding (PCE) as compared to the original SDMD method, averaged per image type.

Type	SDMD + ALE	SDMD + ALE + PCE
(**a1**) Graphics art images	0.0002↓ / 29%↑	0.0010↓ / 74%↑
(**a2**) Cartoon images	0.0006↓ / 38%↑	0.0007↓ / 128%↑
(**a3**) Computer graphics	0.0015↓ / 7%↑	0.0019↓ / 45%↑
(**a4**) Medical images	0.0025↓ / 7%↑	0.0032↓ / 18%↑
(**a5**) SciVis data	0.0024↓ / 9%↑	0.0032↓ / 79%↑

**Table 3:** Running time of four SDMD steps on images of different resolutions, in milliseconds.

Operation	320 × 200	640 × 400	960 × 600	1280 × 800	1600 × 1000	1920 × 1200	2240 × 1400	2560 × 2000
**Skeletonization**	48	182	294	729	1119	1559	3501	4168
**Spline fitting**	1648	1236	2136	2098	2812	3849	4650	5592
**Reconstruction**	62	118	292	561	951	1583	2502	3618
**Interpolation**	28	260	345	1004	1479	2105	3904	4960

## Data Availability

All experiments in this paper, including the original image datasets, SSDMD encoding results, and computed quality metrics, are openly available at [41].
